# DNA Methylation as a Biomarker for Cardiovascular Disease Risk

**DOI:** 10.1371/journal.pone.0009692

**Published:** 2010-03-15

**Authors:** Myungjin Kim, Tiffany I. Long, Kazuko Arakawa, Renwei Wang, Mimi C. Yu, Peter W. Laird

**Affiliations:** 1 Norris Comprehensive Cancer Center, Departments of Surgery and of Biochemistry and Molecular Biology, Keck School of Medicine, University of Southern California, Los Angeles, California, United States of America; 2 Masonic Cancer Center, University of Minnesota, Minneapolis, Minnesota, United States of America; Johns Hopkins University, United States of America

## Abstract

**Background:**

Elevated serum homocysteine is associated with an increased risk of cardiovascular disease (CVD). This may reflect a reduced systemic remethylation capacity, which would be expected to cause decreased genomic DNA methylation in peripheral blood leukocytes (PBL).

**Methodology/Principal Findings:**

We examined the association between prevalence of CVD (myocardial infarction, stroke) and its predisposing conditions (hypertension, diabetes) and PBL global genomic DNA methylation as represented by ALU and Satellite 2 (AS) repetitive element DNA methylation in 286 participants of the Singapore Chinese Health Study, a population-based prospective investigation of 63,257 men and women aged 45–74 years recruited during 1993–1998. Men exhibited significantly higher global DNA methylation [geometric mean (95% confidence interval (CI)): 159 (143, 178)] than women [133 (121, 147)] (*P* = 0·01). Global DNA methylation was significantly elevated in men with a history of CVD or its predisposing conditions at baseline (*P* = 0·03) but not in women (*P* = 0·53). Fifty-two subjects (22 men, 30 women) who were negative for these CVD/predisposing conditions at baseline acquired one or more of these conditions by the time of their follow-up I interviews, which took place on average about 5·8 years post-enrollment. Global DNA methylation levels of the 22 incident cases in men were intermediate (AS, 177) relative to the 56 male subjects who remained free of CVD/predisposing conditions at follow-up (lowest AS, 132) and the 51 male subjects with a diagnosis of CVD or predisposing conditions reported at baseline (highest AS 184) (*P* for trend = 0.0008) No such association was observed in women (*P* = 0.91). Baseline body mass index was positively associated with AS in both men and women (*P* = 0·007).

**Conclusions/Significance:**

Our findings indicate that elevated, not decreased, PBL DNA methylation is positively associated with prevalence of CVD/predisposing conditions and obesity in Singapore Chinese.

## Introduction

Cardiovascular disease (CVD) is the leading cause of death in most countries [1]. Risk factors known to predispose to the development of CVD include increasing age, male gender, diabetes mellitus, high blood cholesterol, tobacco smoking, high blood pressure (hypertension), obesity, physical inactivity, and family history [1]. Elevated plasma homocysteine is also an independent risk factor for CVD [2], [3]. The mechanism by which elevated homocysteine contributes to CVD risk is not well understood, but it is well-established that dietary folate and vitamin B supplementation can reduce serum homocysteine levels by facilitating remethylation of homocysteine to methionine [4], [5], [6]. Accumulation of homocysteine can lead to increased intracellular levels of S-adenosylhomocysteine, a transmethylation inhibitor [7], [8]. The remethylation cycle is essential for the systemic methyl donor supply, which is used for important biological processes, such as cytosine-5 methylation of genomic DNA, an epigenetic modification that plays an important role in maintaining genomic stability, chromatin structure, and in controlling transcriptional capacity [2], [9]. Global DNA hypomethylation has been observed in atherosclerotic lesions as a consequence of low dietary folate or elevated plasma homocysteine in humans and animal models [10], [11]. We have previously identified age, sex, plasma folate, vitamin B-12 and vitamin B-6, and methylenetetrahydrofolate reductase (*MTHFR*) genotype as independent predictors of plasma homocysteine in Singapore Chinese [12]. In this study, we examined the relationships between prevalence of CVD (myocardial infarction, stroke) or its predisposing conditions (hypertension, diabetes) and peripheral blood leukocytes (PBL) global genomic DNA methylation to verify the potential value of DNA methylation as a CVD biomarker, using a validated MethyLight-based assay for ALU and Satellite 2 repetitive element (AS) DNA methylation [13].

## Methods

### Study Population

The subjects were participants of the Singapore Chinese Health Study, a population-based prospective cohort study of Chinese men and women, aged 45–74 years at baseline. They belonged to the two major Chinese dialect groups in Singapore (Hokkien and Cantonese) and lived in government housing estates where 86% of all residents in Singapore resided during the period of enrollment [12]. A total of 63,257 individuals gave informed written consent and enrolled between April 1993 and December 1998. The study was approved by the Institutional Review Boards of the University of Southern California, and the National University of Singapore.

At recruitment, each participant completed an in-person interview using a structured questionnaire that requested information about demographic characteristics, height and weight, use of tobacco, usual physical activity, medical history, and family history of cancer. The questionnaire included a validated semi-quantitative food frequency section listing 165 food items commonly consumed in the study population, from which average daily intake of calories and roughly 100 nutrients and non-nutritive ingredients per subject were computed using the Singapore Food Composition Table [14] which we developed in conjunction with the cohort study.

**Figure 1:**
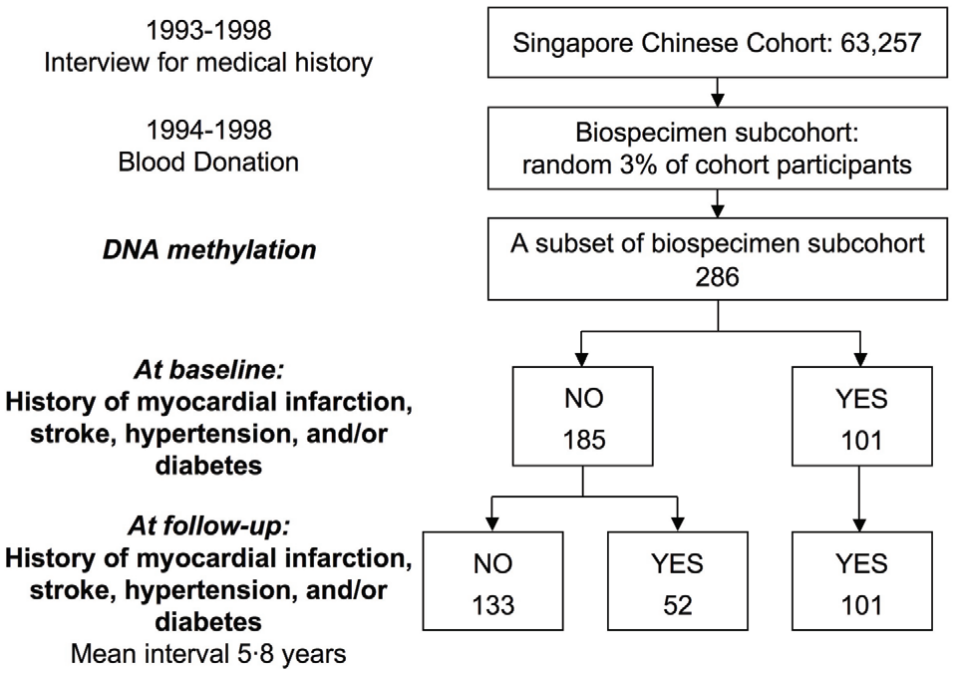
Study Overview.

Between 1994 and 1998, a random 3% sample of cohort participants was recontacted for donation of blood and urine specimens. The 286 subjects in the current study represented the first accrued participants of this biospecimen subcohort [15], [16] (Figure 1). The entire cohort has been continuously followed for the occurrence of incident cancers and deaths ever since. All surviving cohort participants were interviewed by telephone during 1999–2003 for an updated medical history. The mean time interval between the two interviews (baseline and follow-up) is 5·8 years (range, 2·6–11·0 years).

### DNA Methylation Analysis

DNA was extracted from peripheral blood leukocytes collected from the 286 (129 men, 157 women) study subjects. Sodium bisulfite conversion of genomic DNA was conducted. The samples used in our study were stored at −30°C to minimize DNA degradation and (methyl)cytosine deamination. Methylation levels of repetitive elements were determined using MethyLight technology as described previously [13]. The performance characteristics of the MethyLight assay, including precision and reproducibility have been well described [17]. Briefly, bisulfite-to-bisulfite coefficient of variation (CV) of percent of methylated reference (PMR; degree of DNA methylation) ranged from 0·10 to 0·38 (mean, 0·21), and MethyLight run-to-run CV of PMR ranged from 0·046 to 0·60 (mean, 0·31). The MethyLight data specific for methylated repetitive elements were calculated as percent of methylated reference (PMR) using M. SssI-treated DNA as a methylated reference and the ALU-based control reaction (ALU-C4) as a control reaction to measure the levels of input DNA to normalize the signal for each methylation reaction. Thus the PMR can be defined as ((METHYLATED GENE/CONTROL REACTION)_sample_)/((METHYLATED GENE/CONTROL REACTION)_SssI-Reference_)*100, in which “METHYLATED GENE” refers the methylation measurement at a particular locus such as ALU or SAT2 and “CONTROL REACTION” refers to the methylation-independent measurement using the Alu-based control reaction. The composite methylation measurements of ALU (ALU-M2) and SAT2 (SAT2-M1) were used for MethyLight-based estimates of genomic 5-methylcytosine content [13]. The AS index for a given subject is defined as the arithmetic mean of ALU-M2 and SAT2-M1.

### Statistical analysis

Global methylation as assessed by the AS index showed a markedly skewed distribution toward high values. This deviation from normality was largely corrected via a logarithmic transformation of the actual values of AS. Thus, geometric means (as opposed to arithmetic means) of AS and their corresponding 95% confidence intervals were presented. We decided on a more conservative, non-parametric approach to formal statistical testings of the data. Therefore, we employed the generalized linear modeling (GLM) methods on ranked values of the AS index, as opposed to its logarithmically transformed values, in calculating all *P* values reported in this paper. Since length of sample storage may have an influence on our data, we repeated all analyses with length of sample storage (months) as an additional covariate in the GLM models. No material changes to the results are noted. Since serum homocysteine may be a confounder in the examination of AS in relation to BMI and CVD, it was entered as a covariate to the appropriate GLM models in addition to age, gender and BMI. All statistical computations were conducted using the statistical program SAS, version 6·12 (SAS Institute Inc, Cary, NC). All *P* values reported are two-tailed and statistical significance was defined as *P*<0·05.

## Results

### Global DNA methylation and gender

Men had significantly higher AS values within each age group (*P* = 0·02; Table 1), consistent with our recent finding of higher DNA methylation at unique genomic loci in men than in women [15]. However, age, adjusted for gender, was not associated with AS. There is no evidence of an interaction effect of age and gender on AS level. All subsequent statistical analyses were adjusted for age and gender.

**Table 1:** Geometric mean (95% confidence interval)^1^ levels of the AS index according to gender and age at blood draw.

n	Total	n	Men^1^	n	Women^1^
286	144 (135, 155)	129	159 (143, 178)	157	133 (121, 147)
**Age at blood draw (yrs)**					
	55–59	47	155 (131, 183)	66	132 (115, 153)
	60–64	38	171 (141, 206)	34	143 (117, 174)
	65–69	27	136 (109, 171)	27	112 (89, 139)
	70–77	17	178 (135, 236)	30	150 (122, 186)
**p for trend** 1,2 **(age)**		0·82			
**p-value** 1,2 **(gender)**		**0·01**			
**p-value** 1,2 (age*gender)		0·91			

1 From Generalized Linear Model with adjustment for gender and age.

2 Generalized Linear Modeling was performed on ranks (as opposed to actual values) of AS, with adjustment for gender and age. All p-values are two-sided.

### Global DNA methylation and CVD

Subjects with a self-reported history of physician-diagnosed heart attack (myocardial infarction) and/or stroke at baseline showed a borderline (*P* = 0·045) significantly higher mean AS [n = 14; geometric mean (95% confidence interval (CI)): 201 (145, 280)] compared to those without such a history [n = 272; 145 (126, 168)] (Table 2). Subjects with a self-reported history of myocardial infarction, stroke, hypertension and/or diabetes showed a higher mean AS measurement that is of borderline statistical significance (*P* = 0·055) relative to those without such histories [n = 101; 160 (136, 188) vs. n = 185; 138 (118, 162)]. This relationship between prevalence of myocardial infarction, stroke or their predisposing medical conditions and global DNA methylation was principally observed in men (*P* = 0·03). To further delineate whether this gender effect and CVD status were correlated, we analyzed the AS index by gender stratified by CVD status. The gender effect was mostly coming from the subgroup of subjects with a history of CVD at baseline (*P* = 0·007; Table 3).

**Table 2:** Geometric mean (95% confidence interval)^1^ levels of the AS index by selected medical conditions at baseline.

	n	Total subjects	n	Males	n	Females
**Myocardial infarction**						
No	276	147 (127, 169)	123	160 (128, 202)	153	133 (110, 161)
Yes	10	198 (134, 291)	6	267 (155, 460)	4	173 (77, 387)
** p-value** 2		0·15		0·07		0·52
**Stroke**						
No	282	147 (127, 170)	127	163 (129, 205)	155	131 (107, 160)
Yes	4	215 (119, 389)	2	273 (111, 669)	2	140 (112, 175)
** p-value** 2		0·15		0·16		0·41
**Hypertension**						
No	203	140 (120, 164)	90	149 (116, 190)	113	134 (111, 162)
Yes	83	163 (137, 193)	39	191 (146, 251)	44	135 (76, 241)
** p-value** 2		0·07		0·07		0·93
**Diabetes**						
No	255	147 (127, 171)	111	161 (127, 204)	144	136 (111, 167)
Yes	31	152 (121, 191)	18	179 (127, 254)	13	127 (92, 175)
** p-value** 2		0·87		0·47		0·58
**Myocardial infarction and/or stroke**						
No	272	145 (126, 168)	121	160 (127, 201)	151	133 (110, 162)
Yes	14	201 (145, 280)	8	269 (167, 434)	6	146 (91, 234)
** p-value** 2		**0·045**		**0·02**		0·66
**Hypertension and/or diabetes among at-risk subjects** 3						
No	185	139 (118, 164)	78	147 (112, 191)	107	130 (105, 162)
Yes	87	155 (131, 184)	43	179 (137, 234)	44	136 (108, 171)
** p-value** 2		0·19		0·14		0·59
**History of Myocardial infarction, stroke, hypertension or diabetes**						
No	185	138 (118, 162)	78	143 (111, 185)	107	131 (106, 162)
Yes	101	160 (136, 188)	51	187 (145, 241)	50	138 (113, 171)
** p-value** 2		0·055		**0·03**		0·53

1 From Generalized Linear Model with adjustment for age, and gender (in total subjects).

2 Generalized Linear Modeling was performed on ranks (as opposed to actual values) of AS, with adjustment for age, and gender (in total subjects). All p-values are two-sided.

3 Subjects who had a history of myocardial infarction and/or stroke at baseline were deleted from this analysis.

**Table 3:** Geometric means (95% CI)^1^ of the AS index by gender, stratified by CVD status.

CVD^2^ status	Men	Women	p-value^3^
No	143 (111, 185)	131 (106, 162)	0.18
Yes	187 (145, 241)	138 (113, 171)	**0.007**
Total	161 (145, 179)	137 (124, 151)	**0.01**

1 From Generalized Linear Model with adjustment for age and CVD status (for total subjects).

2 CVD is defined as history of myocardial infarction, stroke, hypertension or diabetes.

3 Generalized Linear Modeling was performed on ranks (as opposed to actual values) of AS with adjustment for age and CVD status (for total subjects).

### Global DNA methylation and BMI

The constellation of positive associations between global PBL DNA methylation and prevalence of CVD or its risk factors suggests a potential relationship between metabolic syndrome [18] and global DNA methylation. However, serum triglyceride levels, total cholesterol, high density lipoprotein cholesterol, and low density lipoprotein cholesterol were not correlated with AS (Table 4), in agreement with the finding that DNA methylation of LINE-1 repetitive sequences was not altered in atherosclerosis-prone Apolipoprotein E*-*null aortic DNA compared with controls [10]. Similarly, there were no statistically significant associations between plasma homocysteine, folate, vitamin B12, vitamin B6, and AS levels (Table 4). Furthermore, levels of homocysteine were unrelated to AS, independent of CVD status (Supplementary Table S1). Quartile cut-points of plasma homocysteine, B vitamins and cholesterols were shown in Supplementary Table S2. We then examined the polymorphisms of two folate metabolizing enzymes, *MTHFR and TYMS,* in relation to AS levels. Genotypes of *MTHFR* (*P* = 0.03) but not *TYMS* (*P* = 0.24) were significantly associated with AS levels (Table 4). Meanwhile, baseline body-mass index (BMI) was positively associated with AS (*P* = 0·007; Table 5), consistent with our hypothesis. Subjects with BMI of 24 kg/m^2^ or higher [n = 74; 178 (147, 214)] showed elevated AS compared to those with BMI below 24 kg/m^2^ [n = 212; 140 (121, 163)].

**Table 4:** Geometric means (95% confidence interval)^1^ levels of the AS index by methylation and cholesterol variables at baseline.

	n	Total subjects	n	Males	n	Females
**Homocysteine (umol/L)**						
1st quartile	73	155 (125, 191)	23	165 (115, 236)	50	144 (111, 187)
2nd quartile	73	143 (117, 173)	24	142 (103, 197)	49	138 (108, 176)
3rd quartile	72	146 (122, 175)	37	155 (116, 207)	35	138 (109, 174)
4th quartile	68	153 (127, 184)	45	180 (138, 234)	23	121 (92, 159)
**p for trend** 2		0·68		0·62		0·26
**Folate (nmol/L)**						
1st quartile	67	142 (118, 171)	44	152 (116, 199)	23	136 (103, 180)
2nd quartile	67	175 (144, 214)	30	175 (12, 240)	37	173 (134, 224)
3rd quartile	70	145 (120, 174)	31	176 (131, 237)	39	121 (95, 154)
4th quartile	74	143 (118, 173)	24	160 (114, 225)	50	128 (101, 161)
**p for trend** 2		0·65		0·42		0·15
Missing	8		0		8	
**Vitamin B-12 (pmol/L)**						
1st quartile	68	139 (115, 167)	39	143 (108, 188)	29	135 (103, 178)
2nd quartile	71	160 (133, 194)	33	192 (145, 253)	38	134 (103, 174)
3rd quartile	68	135 (111, 165)	36	141 (104, 190)	32	129 (99, 169)
4th quartile	72	158 (131, 190)	21	187 (133, 263)	51	138 (109, 173)
**p for trend** 2		0·51		0·34		0·94
Missing	7		0		7	
**Vitamin B-6 (nmol/L)**						
1st quartile	66	138 (114, 166)	40	147 (111, 197)	26	127 (98, 166)
2nd quartile	69	139 (115, 168)	31	150 (109, 208)	38	127 (100, 161)
3rd quartile	71	162 (134, 197)	30	178 (132, 241)	41	148 (114, 192)
4th quartile	73	157 (130, 190)	23	175 (126, 242)	50	142 (112, 180)
**p for trend** 2		0·07		0·16		0·23
Missing	7		5		2	
**Summed quartile ranks**						
**(Folate + VB-12 + VB-6)**						
0 – 2	56	131 (107, 160)	37	145 (108, 195)	19	116 (85, 158)
3 – 4	78	164 (137, 198)	41	175 (131, 234)	37	156 (121, 201)
5 – 6	73	144 (119, 175)	29	158 (117, 213)	44	131 (102, 168)
7 – 9	64	153 (124, 188)	17	197 (136, 286)	47	130 (101, 168)
**p for trend** 2		0·42		0·15		0·79
Missing	15		5		10	
**MTHFR**						
AA	168	157 (134, 183)	76	178 (140, 228)	92	139 (113, 171)
AV	91	133 (112, 159)	39	144 (109, 191)	52	123 (99, 154)
VV	24	133 (101, 177)	13	137 (91, 206)	11	132 (89, 197)
**p for trend** 2		**0·03**		**0·03**		0·32
Missing	3		1		2	
**TS**						
3/3	199	152 (131, 176)	91	166 (131, 210)	108	138.3 (114, 168)
Other	87	137 (114, 165)	38	156 (116, 211)	49	120.4 (95, 153)
**p-value** 2		0·24		0·78		0·18

	n	Total subjects	n	Males	n	Females
**Total cholesterol**						
1st quartile	70	147 (120, 179)	41	138 (102, 186)	29	159 (122, 209)
2nd quartile	71	144 (121, 171)	37	175 (135, 227)	34	115 (90, 146)
3rd quartile	74	162 (134, 196)	24	180 (128, 252)	50	145 (115, 183)
4th quartile	71	144 (119, 175)	27	151 (108, 212)	44	133 (106, 168)
**p for trend** 2		0·81		0·35		0·59
**HDL**						
1st quartile	72	154 (128, 185)	42	165 (126, 216)	30	141 (109, 182)
2nd quartile	71	143 (119, 171)	37	148 (112, 197)	34	134 (106, 170)
3rd quartile	76	146 (120, 177)	32	193 (143, 260)	44	112 (87, 144)
4th quartile	67	152 (124, 185)	18	142 (98, 205)	49	142 (112, 179)
**p for trend** 2		0·93		0·59		0·71
**LDL**						
1st quartile	67	148 (122, 180)	33	142 (105, 193)	34	154 (119, 199)
2nd quartile	70	151 (125, 183)	41	172 (130, 227)	29	132 (101, 172)
3rd quartile	71	143 (118, 173)	26	165 (119, 230)	45	126 (99, 160)
4th quartile	69	142 (117, 172)	23	168 (119, 237)	46	125 (99, 158)
**p for trend** 2		0·42		0·38		0·07
Missing	9		6		3	
**Triglyceride**						
1st quartile	73	140 (115, 170)	26	147 (108, 199)	47	129 (100, 166)
2nd quartile	73	140 (117, 169)	42	170 (128, 226)	31	113 (87, 146)
3rd quartile	69	152 (126, 184)	28	174 (128, 236)	41	135 (107, 172)
4th quartile	71	159 (132, 191)	33	166 (121, 229)	38	151 (120, 191)
**p for trend** 2		0·14		0·55		0·14

1 From Generalized Linear Model with adjustment for age, and gender (in total subjects).

2 Generalized Linear Modeling was performed on ranks (as opposed to actual values) of AS, with adjustment for age, and gender (in total subjects). All p-values are two-sided.

*Quartile cut-points of plasma homocysteine, B vitamins and cholesterols were shown in Supplementary Table S2.

**Table 5:** Geometric mean (95% confidence interval)^1^ levels of the AS index according to baseline BMI and subjects' history of myocardial infarction, stroke, hypertension and/or diabetes at baseline and at follow-up/death.

BMI at baseline	Myocardial infarction, stroke, hypertension and diabetes at baseline	Myocardial infarction, stroke, hypertension and diabetes at follow-up/death	n	Mean AS at baseline Total subjects	n	Mean AS at baseline Males	n	Mean AS at baseline Females
<24			212	140 (121, 163)	93	152 (119, 194)	119	126 (103, 154)
24+			74	178 (147, 214)	36	189 (139, 256)	38	160 (126, 204)
**p value** 2 **(BMI)**				**0·007**		**0·04**		0·07
	No	No	133	139 (117, 165)	56	132 (101, 173)	77	138 (110, 173)
	No	Yes	52	140 (113, 174)	22	177 (126, 250)	30	114 (86, 151)
	Yes^3^	Yes^3^	101	161 (136, 189)	51	184 (141, 240)	50	139 (112, 172)
**p for trend** 2 **(myocardial infarction, stroke, hypertension, diabetes)**				0·11		**0·008**		0·91
<24	No	No	111	138 (116, 164)	48	133 (102, 175)	63	136 (108, 171)
<24	No	Yes	38	130 (103, 165)	15	163 (111, 239)	23	107 (80, 145)
<24	Yes	Yes	63	139 (115, 168)	30	162 (120, 220)	33	119 (93, 151)
24+	No	No	22	139 (104, 186)	8	109 (66, 181)	14	144 (102, 206)
24+	No	Yes	14	166 (119, 232)	7	199 (119, 335)	7	134 (86, 208)
24+	Yes	Yes	38	202 (162, 252)	21	217 (156, 303)	17	185 (137, 251)

1 From Generalized Linear Model with adjustment for age, gender (in total subjects), and serum homocysteine.

2 Generalized Linear Modeling was performed on ranks (as opposed to actual values) of AS, with adjustment for age, gender (in total subjects), and serum homocysteine. All p-values are two-sided.

3 ICD9 codes on death certificates are:

Myocardial infarction = 402 (hypertensive heart disease), 410 (acute myocardial infarction), 411 (other acute and subacute ischemic heart disease), 412 (old myocardial infarction), 413 (angina), 414 (other forms of chronic heart disease), 427 (cardiac dysrhythmia), and 428 (heart failure).

Stroke = 430–438.

Diabetes = 250.

Hypertension = 401 (essential or primary hypertension), 402 (hypertensive heart disease), 403 (hypertensive renal disease), 404 (hypertensive heart and renal disease), and 405 (secondary hypertension).

### Global DNA methylation and CVD at follow-up

We explored the association between CVD or predisposing conditions and global DNA methylation in more detail by analyzing newly diagnosed cases at follow-up among the 185 subjects free of CVD or predisposing conditions at the time of the baseline interview. All cohort participants were interviewed by telephone during 1999–2003 for an updated medical history (The Follow-up I Survey). The mean time interval between the two interviews (baseline and follow-up I) was 5·8 years (range, 2·6–11·0 years) among the 52,325 participants of the Follow-up I Survey. We identified 47 subjects who were free of a history of myocardial infarction, stroke, hypertension, and/or diabetes at recruitment but had developed at least one of these conditions during follow-up. In addition, we identified from death certificate reviews that five subjects had died of one of these listed conditions as of December 31, 2004. Meanwhile, 133 subjects were still negative for CVD or its predisposing conditions by the time of their follow-up interviews. Male (*P* = 0.008) but not female (*P* = 0.91) subjects exhibited an association between AS levels and status of CVD/predisposing conditions at baseline and at followup/death (Table 5). Among men, the 22 incident cases [177 (126, 250)] exhibited higher levels of AS relative to the 56 subjects [132 (101, 173)] without any of these medical conditions both at baseline and at follow-up. The highest levels of AS were observed among the 51 subjects [184 (141, 240)] who already were positive for these medical conditions at recruitment. When BMI and medical history were examined in combination with respect to AS, the highest levels of AS were noted among subjects who, at baseline, possessed the highest level of BMI (24 kg/m^2^ and above) and a physician-diagnosed history of myocardial infarction, stroke, hypertension and/or diabetes (Table 5), although the small number of subjects in each cell precludes firm conclusions.

## Discussion

We present here the results of a population-based prospective cohort study of risk factors for CVD. This study was initiated to ascertain whether PBL DNA methylation could serve as a stable measure of systemic methyl group supply, analogous to the use of glycated forms of hemoglobin to provide measures of long-term mean blood glucose levels. However, we did not observe the anticipated correlations between PBL DNA methylation and plasma folate and homocysteine, dietary folate and the B vitamins, and folate metabolizing genotypes such as *TYMS*. Furthermore, we noted a statistically significant, positive association between PBL DNA methylation and prevalence of CVD or its risk factors, primarily in men, when we had anticipated an inverse association between the two sets of factors. This suggests that, rather than folate insufficiency, a different mechanism, such as systemic inflammation, may lead to increased PBL DNA methylation. Our results differ from those of Castro et al. (2003), who found that vascular disease patients with elevated plasma tHcy and AdoHcy concentrations and low plasma AdoMet/AdoHcy ratios had lower levels of genomic DNA methylation [19]. However, this study was based on a very small sample size of 17 vascular disease cases and 15 controls. Although Castro et al. (2003) used the intracellular AdoMet/AdoHcy ratio as a predictor of cellular methylation capacity, they failed to observe such association in their study. The global DNA methylation status and homocysteine, both plasma tHcy and AdoHcy, also seemed to be less correlated each other (*r* = 0.47; *r* = 0.54, respectively). Moreover, it has been observed that imprinted gene H19 is hypermethylated, not hypomethylated, in brain and aorta of hyperhomocysteinemic mice, although the effect of hyperhomocysteinemia on H19 DMD methylation was tissue-specific in these mice [20]. The result of significantly higher AS in men is consistent with our recent finding of higher DNA methylation at unique genomic loci in men than in women [15]. It has been reported that global DNA methylation levels decrease with age [21], [22]. However, the relatively narrow elderly age range (55–77 years) of our study population at blood draw may account for the lack of association between age and AS in this study. Age-dependent decrease of global DNA methylation levels measured by HPLC was also fairly small in human PBL among age groups [22].

Although we did not find a link between lipid metabolites [18] and global DNA methylation, baseline BMI was positively associated with AS. High relative weight is considered a risk factor for CVD in both western [23] and Chinese [24] populations. It is worth noting that most diabetics among Chinese have normal BMI according to western standards [25] and the recognized cutpoint for at-risk Chinese is BMI of 24 kg/m^2^ [26] or higher.

In summary, this is the first report of an association between global DNA methylation assessed by ALU/SAT2 methylation [13] and prevalence of CVD, in addition to its risk factors, including male gender and obesity in a population-based Singapore Chinese cohort, a relatively lean population. Our novel findings, derived from analysis that were exploratory in nature, require confirmation from studies on other Asians as well as more distinct ethnic groups, such as those in the West with higher BMI. If confirmed, this blood-based marker could offer exciting new opportunities for population-based CVD risk assessment and prevention.

## Supporting Information

Table S1 Geometric means (95% CI) of AS index by serum homocysteine at baseline and gender, stratified by CVD status.
(0.05 MB DOC)

Table S2 Quartile cut-points of plasma homocysteine, B vitamins and cholesterols.
(0.04 MB DOC)
