# Supplementary material for: DNA Methylation as a Biomarker for Cardiovascular Disease Risk
Source: PLoS One. 2010 Mar 15;5(3):e9692. doi: 10.1371/journal.pone.0009692 (PMC2837739; doi:10.1371/journal.pone.0009692)
Supplement: Table S1 — Geometric means (95% CI) of AS index by serum homocysteine at baseline and gender, stratified by CVD status. (0.05 MB DOC) [file pone.0009692.s001.doc]

**Supplementary Table S1.** Geometric means (95% CI)^1^ of AS index by serum homocysteine at baseline and gender, stratified by CVD status

|  | **n** | **Total subjects** | **n** | **Males** | **n** | **Females** |
| --- | --- | --- | --- | --- | --- | --- |
| **CVD**^2^ **(-)**  **Homocysteine (umol/L)** |  |  |  |  |  |  |
| 1st quartile | 51 | 145 (110, 190) | 17 | 139 (86, 226) | 34 | 143 (101, 203) |
| 2nd quartile | 52 | 144 (112, 186) | 16 | 123 (81, 191) | 36 | 153 (112, 210) |
| 3rd quartile | 45 | 141 (111, 178) | 21 | 139 (92, 211) | 24 | 135 (100, 181) |
| 4th quartile | 37 | 139 (107, 181) | 24 | 143 (95, 215) | 13 | 121 ( 82, 179) |
| **p for trend^3^** |  | 0·57 |  | 0·97 |  | 0·37 |
| **CVD**^2^ **(+)**  **Homocysteine (umol/L)** |  |  |  |  |  |  |
| 1st quartile | 22 | 178 (128, 249) | 6 | 239 (129, 444) | 16 | 139 (92, 209) |
| 2nd quartile | 21 | 133 (98, 181) | 8 | 183 (109, 308) | 13 | 104 (70, 153) |
| 3rd quartile | 27 | 152 (116, 199) | 16 | 181 (118, 275) | 11 | 139 (95, 204) |
| 4th quartile | 31 | 170 (131, 220) | 21 | 237 (161, 348) | 10 | 118 (80, 173) |
| **p for trend^3^** |  | 0·75 |  | 0·55 |  | 0·52 |

^1^ From Generalized Linear Model with adjustment for age, and gender (in total subjects).

^2^ CVD is defined as history of myocardial infarction, stroke, hypertension or diabetes.

^3^ Generalized Linear Modeling was performed on ranks (as opposed to actual values) of AS with adjustment for age and gender (in total subjects); all p-values are two-sided.
