# Supplementary material for: DNA Methylation as a Biomarker for Cardiovascular Disease Risk
Source: PLoS One. 2010 Mar 15;5(3):e9692. doi: 10.1371/journal.pone.0009692 (PMC2837739; doi:10.1371/journal.pone.0009692)
Supplement: Table S2 — Quartile cut-points of plasma homocysteine, B vitamins and cholesterols. (0.04 MB DOC) [file pone.0009692.s002.doc]

**Supplementary Table S2.** Quartile cut-points of plasma homocysteine, B vitamins and cholesterols

|  | **Quartiles** | | | |
| --- | --- | --- | --- | --- |
|  | **1st** | **2nd** | **3rd** | **4th** |
| **Homocysteine (umol/L)** |  ≤8.71 | 8.72~≤10.54 | 10.55~≤12.74 | 12.75+ |
|  |  |  |  |  |
| **Folate (nmol/L)** | ≤9.99 | 10.00~≤13.64 | 13.65~≤19.34 | 19.35+ |
|  |  |  |  |  |
| **Vitamin B-12 (pmol/L)** | ≤245.63 | 245.64~≤322.78 | 322.79~≤410.04 | 410.05+ |
|  |  |  |  |  |
| **Vitamin B-6 (nmol/L)** | ≤21.09 | 21.10~≤33.29 | 33.30~≤51.24 | 51.25+ |
|  |  |  |  |  |
| **Total cholesterol (mmol/L)** | ≤4.95 | 4.96~≤5.75 | 5.76~≤6.39 | 6.40+ |
|  |  |  |  |  |
| **HDL (mmol/L)** | ≤1.11 | 1.12~≤1.31 | 1.32~≤1.54 | 1.55+ |
|  |  |  |  |  |
| **LDL (mmol/L)** | ≤2.86 | 2.87~≤3.48 | 3.49~≤4.12 | 4.13+ |
|  |  |  |  |  |
| **Triglyceride (mmol/L)** | ≤1.23 | 1.24~≤1.68 | 1.69~≤2.35 | 2.36+ |
